# Effects of tea oil camellia (*Camellia oleifera* Abel.) shell-based organic fertilizers on the physicochemical property and microbial community structure of the rhizosphere soil

**DOI:** 10.3389/fmicb.2023.1231978

**Published:** 2023-08-10

**Authors:** Anxiang Huang, Zhongwei Wang, Dingyun Yang, Shoulu Yang, Wennian Bai, Nengying Wu, Xiang Lu, Zhu Liu

**Affiliations:** ^1^Guizhou Academy of Forestry, Guiyang, China; ^2^Qianxinan Ecological Environment Monitoring Centre, Xingyi, China

**Keywords:** microbial community structure, organic fertilizer, rhizosphere soil, soil porosity, tea oil camellia shell

## Abstract

Soil microorganisms play important roles in promoting soil ecosystem restoration, but much of the current research has been limited to changes in microbial community structure in general, and little is known regarding the soil physicochemical property and microbial community structure. In this study, four organic fertilizers were first prepared based on tea oil camellia shell (TOCS). Our findings indicate that the application of BOFvo increased both total pore volume and BET surface area of the rhizosphere soils, as well there was a remarkable enhancement in total organic matter (TOM), total nitrogen (TN), available nitrogen (AN), total phosphorus (TP), total potassium (TK), and available potassium (AK) contents of the rhizosphere soils. Meanwhile, in comparison to the CK and CF groups, the utilization of BOFvo led to a substantial increase in both average yield and fruiting rate per plant at maturity, as well resulted in a significant increase in TN and TP contents of tea oil camellia leaves. Furthermore, our findings suggest that the application of TOCS-based organic fertilizers significantly enhances the microbial diversity in the rhizosphere soils with Proteobacteria and Ascomycota being the dominant bacterial and fungal phyla, respectively, and *Rhodanobacter* and *Fusarium* being the dominant bacterial and fungal genus, respectively. Redundancy analysis (RDA) indicates that the physicochemical characteristics of TOCS-based organic fertilizers had a significant impact on the composition and distribution of microbial communities in the rhizosphere soils. This study will facilitate the promotion and application of TOCS-based organic fertilizers, thereby establishing a foundation for the reuse of tea oil camellia waste resources.

## 1. Introduction

With the expansion of the economy and population, coupled with the acceleration of modern industrialization, there has been a surge in global energy demand (Belaïd et al., 2023). However, due to the scarcity of non-renewable fossil fuels and the increasingly severe problem of environmental pollution, the development of renewable energy has become a mainstream trend. Biomass accounts for a significant portion of renewable energy sources, and in the near future, it will be the most widely used form of renewable energy (Popp et al., 2021). As a result, re-searchers have paid increasing attention to the development of alternative fuels and renewable energy sources. In 2019, the social capital invested 7 billion Chinese Yuan (CNY) in the camellia industry development (Zhang J. et al., 2018). Over the next few years, central financial funds will prioritize supporting the transformation of low-quality camellia forests, and technology research and development for the camellia industry will be included in China’s national “14th Five-Year Plan” for science and technology (Zhu et al., 2013; Hu et al., 2015).

In recent decades, significant amounts of chemical fertilizers have been applied to cultivated fields in order to optimize crop yields and prevent global food shortages (Savci, 2012; Sun et al., 2015). However, the excessive use of traditional chemical fertilizers has significantly impacted the structure of soil microbial community, resulting in nitrogen leaching and compaction, reduced soil organic matter content, and caused other serious degradation that ultimately reduces crop yields (Lauber et al., 2009; Lu et al., 2019; Huang et al., 2022). Consequently, it is crucial to implement novel strategies such as the adoption of organic fertilizers to mitigate the adverse effects of prolonged fertilizer application. The sustainable development of agriculture hinges on appropriate fertilization methods that can enhance crop yields (Chen et al., 2018; Oladele et al., 2019). Previous studies have demonstrated that the utilization of organic fertilizer can significantly enhance soil nutrient availability (such as alkaline N, and available P, K, Fe, Mn, Cu, and Zn), enzyme activities (such as urease, invertase, catalase, and phosphatase), alter microbial community structure and mitigate soil acidification (Samuel et al., 2018; Sheoran et al., 2019; Anyega et al., 2021; Liu et al., 2021).

Tea oil camellia (*Camellia oleifera* Abel.), a distinctive industrial tree species belonging to the genus *Camellia* of the Theaceae family, is extensively cultivated throughout southern and southeastern Asia. In recent years, China has harvested 2.4 million tons of tea oil camellia fruit from 4.4 million hectares of plantations, resulting in significant amounts of solid wastes such as tea oil camellia shell (TOCS) and tea oil camellia cake (TOCC) (Ye et al., 2020). However, owing to the dearth of feasible reuse techniques, TOCS and TOCC are predominantly subjected to incineration or landfill disposal. This leads to diverse types of pollution including groundwater and soil contamination along with greenhouse gas emissions (Su et al., 2014; Qin et al., 2018). Some previous findings have indicated that TOCS and TOCC consist of hemicellulose, cellulose, polysaccharides, and bioactive compounds, which may possess the potential to produce ethanol, vanillin, and activated carbon, additionally, they also contain a variety of macro and trace elements (Zhang L. X. et al., 2018; Zhang et al., 2020; Tang et al., 2021). Thus, it is postulated that TOCS and TOCC could serve as an appealing source of natural and ecofriendly organic fertilizer derived from plant-based raw materials.

The soil microbial community is believed to play a crucial role in maintaining soil health and suppressing plant diseases through various biological processes (Garbeva et al., 2004). Studies have demonstrated that a reduction in the diversity of soil microorganisms can lead to the emergence of soil-borne plant diseases (Thirup et al., 2003; Mazzola, 2004). There is significant interest in monitoring changes to soil microbial community following the application of bio-organic fertilizer. Despite numerous studies on changes in soil microbial community structure, there exists a dearth of research on the formulation of TOCS-based organic fertilizers and their impact on the physicochemical properties and microbial community structure of rhizosphere soil. In terms of fertilization, previous studies have demonstrated that the conversion of TOCS into organic fertilizer through fermentation not only enhances soil nutrition but also addresses issues arising from chemical fertilizer usage and promotes the recycling of renewable resources (Hu et al., 2019; Yang et al., 2019).

Based on this, the objective of this study was to prepare TOCS-based organic fertilizers and investigate their impact on the soil physicochemical property and microbial community structure in the rhizosphere soil as well as both average yield and fruiting rate per plant at maturity and nutrient contents in tea oil camellia leaves.

## 2. Materials and methods

### 2.1. Materials collection

Tea oil camellia shell and TOCC were collected from Ceheng County (with an altitude of 664 m, north latitude of 24°59′55.05′, and east longitude of 105°47′14.05″), Qianxinan Prefecture, Guizhou Province, China. Decomposition agents were purchased from South China State Farming Agricultural Technology Development Co. Ltd. (Chengdu, China). Microbial agents were purchased from Shanghai Jiabosen Bioengineering Co., Ltd. (Shanghai, China). Commercial compound fertilizer [CF; total nitrogen (TN) content 20%, total phosphorus (TP) content 10%, and total potassium (TK) content 10%] was purchased from Guizhou Xiyang Industrial Co., LTD (Guiyang, China).

### 2.2. Preparation of pre-fermented TOCC

The TOCC underwent a 10-day pre-fermentation process with 3% starter culture and 60% water at 28°C. Upon reaching full mycelial growth, the preparation of pre-fermented TOCC (PTOCC) was subsequently dried for future use.

### 2.3. Preparation of TOCS-based organic fertilizers

In this study, four TOCS-based organic fertilizers (labeled as BOF, BOFvo, OFcsc, and OFpd) were prepared used the reported methods with some modifications (Franke-Whittle et al., 2014; Lu et al., 2022; Chung et al., 2023). Composting aerobic fermentation experiments were conducted from June to August 2021 at the Guizhou Academy of Forestry, Nanming District, Guiyang City, Guizhou Province, China. As shown in Table 1, this study has implemented four treatments, each of which was replicated three times: (1) BOF: 80% of TOCS (partical size ≤5 mm) + 15% of PTOCC (partical size ≤2 mm) + 2.9% of urea (to adjust the initial compost C/N ratio to 32/1) + 0.1% of KH_2_PO_4_ + 2% of decomposition agent; (2) BOFvo: 80% of TOCS (partical size ≤5 mm) + 15% of PTOCC (partical size ≤ 2 mm) + 2.9% of urea + 0.1% of KH_2_PO_4_ + 2% of decomposition agent, with an addition of 3% microbial agent after complete fermentation of BOF; (3) OFcsc: 80% of TOCS (partical size ≤5 mm) + 15% of TOCC (partical size ≤2 mm) + 2.9% of urea + 0.1% of KH_2_PO_4_ + 2% of decomposition agent; (4) OFpd: 80% of TOCS (partical size ≤5 mm) + 15% of poultry dung (instead of pre-fermented TOCC to explore the effects of physicochemical properties and microbial communities on organic fertilizers by adding animal manure) + 2.9% of urea + 0.1% of KH_2_PO_4_ + 2% of decomposition agent. All the raw materials of each treatment were thoroughly mixed and then 50% water was added for moisture control. Subsequently, a plastic film was applied to the surface of each treatment to prevent moisture from evaporating during compost fermentation. The DTSW-2-A digital electronic thermometer (Taian Detu Automation Instrument Co., Ltd., Taian, China) was utilized to measure the temperature of each treatment. Once the temperature exceeded 55°C, the compost piles were turned weekly and left for 1 month until they were fully decomposed.

**TABLE 1:** The composition of raw materials in TOCS-based organic fertilizers.

Treatments	Raw materials contents (%)							
	TOCS	TOCC	PTOCC	Poultry dung	Urea	KH_2_PO_4_	Decomposition agent	Microbial agent
BOF	80	–	15	–	2.9	0.1	2	–
BOFvo	80	–	15	–	2.9	0.1	2	3^a^
OFcsc	80	15	–	–	2.9	0.1	2	–
OFpd	80	–	–	15	2.9	0.1	2	–

^a^Incorporation of 3% microbial agents post-complete fermentation of BOF.

### 2.4. Physicochemical property analysis of TOCS-based organic fertilizers

After fully decomposition, the physicochemical properties of BOF, BOFvo, OFcsc, and OFpd were analyzed using previously established methods with some modifications (Chu and Grogan, 2010). Briefly, pH values were measured using a PH3110 pH meter (WTW Xylem Inc., Munich, Germany) at a ratio of 1:10 [weight (g)/volume (ml)] for each TOCS-based organic fertilizer compared to distilled water. Total organic matter (TOM) content was determined through the wet oxidation method with some modifications (Nieuwenhuize et al., 1994). The contents of TN, TP, and TK were quantified using the Agricultural Industry Standard of the People’s Republic of China (Organic Fertilizer, NY/T 525-2021). The colony-forming units (CFUs) were quantified using a biological microscope (Shanghai Optical Instrument Factory Co., Ltd., Shanghai, China) (Sieuwerts et al., 2008). The unfermented TOCS was used as a negative control (CK). Each treatment was repeated three times.

### 2.5. Soil sample collection and physicochemical properties analysis

**FIGURE 1:**
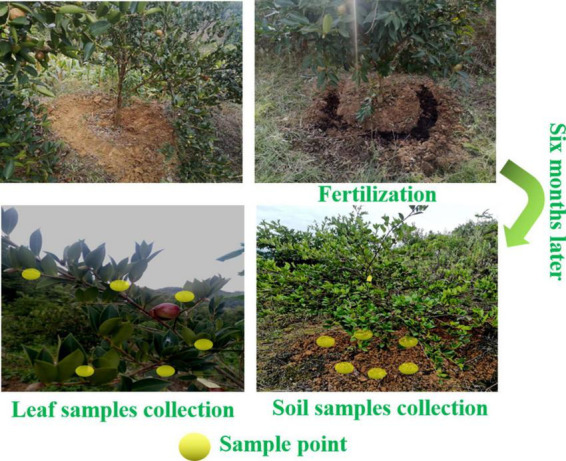
Field trials design as well as soil and leaf samples collection model.

The field trials of four TOCS-based organic fertilizers were conducted in a tea oil camellia forest located in Ceheng County (mean annual precipitation of 1,340.7 mm/a, mean temperature of 19.2°C), Qianxinan Prefecture, Guizhou Province, China. As illustrated in Figure 1, in January 2022, a total of 2 kg of TOCC-based organic fertilizers or CF were applied to each tea oil camellia tree by mixing them with the topsoil and backfill at a depth of 10–20 cm. The experimental period lasted from January to July 2022, during which, 3 parallel soil samples (6 points per replicate) were collected in July 2022 at a depth of 0–20 cm surrounding the lateral roots of each tea oil camellia tree at the fertilization position (Figure 1). The soil samples were promptly homogenized, mixed, and preserved in an on-site refrigerator before being expeditiously transported to our laboratory for subsequent analysis. CF was used as a positive control, whereas no application of different TOCS-based organic fertilizers or CF was used as a negative control (CK).

The rhizosphere soil porosities of tea oil camellia treated with different TOCC-based organic fertilizers or CF were measured using the micromeritics ASAP 2460 version 3.01 (Micromeritics Instrument Ltd., GA, USA). To gain a comprehensive understanding of soil porosity size distribution, the soil porosity diameters were categorized into five groups: crytopores (0.007–0.1 μm), ultramicropores (0.1–5 μm), micropores (5–30 μm), mesopores (30–75 μm), and macropores (>75 μm) (Cameron and Buchan, 2006; Lu et al., 2014). pH values were measured using a PH3110 pH meter (WTW Xylem Inc., Munich, Germany) at a ratio of 1:10 [weight (g)/volume (ml)] for each soil samples compared to distilled water. TOM content was determined through the wet oxidation method with some modifications (Nieuwenhuize et al., 1994). The contents of TN, available nitrogen (AN), TP, available phosphorus (AP), TK, and available potassium (AK) were quantified using the Forestry Industry Standard of the People’s Republic of China (LY/T1228-2015, LY/T1232-2015, and LY/T1234-2015). Each treatment was repeated for three times.

### 2.6. Leaf sample collection and nutrient contents analysis

In July 2022, 3 parallel tea oil camellia leaf samples (6 points per replicate) were collected pre- and post-fertilization for each treatment (Figure 1). The leaf samples were promptly homogenized, mixed, and stored in an on-site refrigerator before being expeditiously transported to our laboratory for analysis of their physicochemical properties. After that, the TN, TP, and TK contents of tea oil camellia leaves were quantified according to the assessment standard in LY/T 1271-1999. Each treatment was repeated for three times.

### 2.7. Average yield and fruiting rate analysis

In this study, the effects of different TOCS-based organic fertilizers on fruit yields of tea oil camellia were investigated by determining the average yield and fruiting rate of individual plants at the mature stage. Subsequently, 10 tea oil camellia trees treated with different TOCC-based organic fertilizers or CF were selected for each treatment and the average yield and fruiting rate of each individual plant were determined at the mature stage using the following formulas, where m is the weight of each camellia fresh fruits, n is the number of camellia flowers of each individual plant, and N is the number of camellia fresh fruits of each individual plant.


$$\text{Average}\,\text{yield}\,\left(\mathrm{kg}\right)=\frac{{\text{m}}_{1}+{\text{m}}_{2}+{\text{⋯m}}_{10}}{10}\times 100\%$$

$$\mathrm{Fruiting}\mathrm{rate}\left(\%\right)=\frac{{\text{N}}_{1}+{\text{N}}_{2}+{\text{⋯N}}_{10}}{{\text{n}}_{1}+{\text{n}}_{2}+{\text{⋯n}}_{10}}\times 100\%$$

### 2.8. High-throughput sequencing and data analysis

The genomic deoxyribonucleic acid (DNA) was extracted from the rhizosphere soil samples (approximately 2.0 g per sample) treated with BOF, BOFvo, OFcsc, and OFpd using a TGuide S96 Magnetic Soil and Stool DNA Kit (Tiangen Biotech Co., Ltd, Beijing, China) according to the manufacturer’s instructions. The DNA quality was assessed by running it on 1% agarose gels prior to its utilization as a template for PCR amplification targeting specific sequencing regions with the corresponding bacterial (341F: 5′-ACTCCTACGGGAGGCAGCA-3′ and 806R: 5′-GGACTACHVGGGTWTCTAAT-3′) and fungal (ITS2F: 5′-GCATCGATGAAGAACGCAGC-3′ and ITS2R: 5′-TCCTCCGCTTATTGATATGC-3′) primers. The PCR products obtained were subjected to sequencing using Illumina HiSeq™ 2000 (Illumina Inc., San Diego, CA, USA). The resulting raw reads underwent filtration with Trimmomatic v0.33 software, followed by primer sequence identification and removal using Cutadapt 1.9.1 software, resulting in clean reads devoid of primer sequences. Subsequently, Usearch v10 software was employed for overlap stitching of the clean reads, which were then filtered based on length range specific to different regions. Finally, the dada2 method implemented in QIIME2 2020.6 software was employed to eliminate the denoised and chimera sequences, resulting in the acquisition of non-chimeric reads.

### 2.9. Statistical analyses

The Vegan package in R language was utilized to analyze the relative abundance of operational taxonomic units (OTUs) and characterize soil microorganism diversity by calculating Shannon, ACE, and Chao1 indices. Meanwhile, principal component analysis (PCA) and heatmaps were utilized via the MetaboAnalyst website^1^ to assess dissimilarities among different TOCS-based organic fertilizers treatments. Redundancy analysis (RDA) was employed in CANOCO5.^2^ In addition, the experimental data mentioned in this article were analyzed using IBM SPSS Statistics software version 20 (IBM SPSS Inc., Chicago, IL, USA) with ANOVA followed by Duncan’s LSD test (*p* < 0.05).

## 3. Results

### 3.1. Physicochemical properties of different TOCS-based organic fertilizers

The physicochemical property results of different TOCS-based organic fertilizers are summarized in Table 2. Intriguingly, compared to the CK group (unfermented TOCS), BOFvo could significantly increase the contents of TOM, TN, TP, and TK by 115.5, 13,492.6, 3,954.8, and 408.0%, respectively, indicating that incorporating a 3% microbial agent after complete fermentation potential can improve the quality of TOCS-based organic fertilizer.

**TABLE 2:** Physicochemical properties of different TOCS-based organic fertilizers.

Parameters	CK (unfermented TOCS)	BOF	BOFvo	OFcsc	OFpd
pH	7.02 ± 0.02b	7.20 ± 0.06b	6.96 ± 0.08b	7.34 ± 0.09a	7.06 ± 0.07b
TOM (%)	32.25 ± 2.07d	66.93 ± 1.45b	69.49 ± 0.81a	33.83 ± 1.60d	60.92 ± 0.29c
TN (%)	0.027 ± 0.008c	4.06 ± 0.08a	3.67 ± 0.06b	4.10 ± 0.06a	3.73 ± 0.09b
TP (%)	0.073 ± 0.004d	3.98 ± 0.16a	2.96 ± 0.10c	3.95 ± 0.08a	3.61 ± 0.07b
TK (%)	2.89 ± 0.011c	12.07 ± 0.34b	14.68 ± 0.04a	12.25 ± 0.37b	12.13 ± 0.12b
CFU (million/g)	0.00 ± 0.00d	4.00 ± 0.04b	12.17 ± 0.15a	0.05 ± 0.01c	0.14 ± 0.02c

Different lowercase letters accompanying the values indicate statistically significant variations (*p* < 0.05) in physicochemical properties among different TOCS-based organic fertilizers.

### 3.2. Physicochemical properties analysis of rhizosphere soils

The impact of different TOCS-based organic fertilizers on the pore characteristics of the rhizosphere soils is presented in Table 3. Results indicate that, compared to the CK and CF groups, the addition of TOCS-based organic fertilizers did not significantly affect the average pore diameter of rhizosphere soils. However, when compared to the CK and CF groups, the addition of TOCS-based organic fertilizers, particularly with BOFvo treatment, significantly increased both total pore volume and BET surface area of the rhizosphere soils by 41.00, 14.29 and 39.14, 33.33%, respectively.

**TABLE 3:** Pore characteristics of rhizosphere soils application of different TOCS-based organic fertilizers.

Parameters	CK	CF	BOF	BOFvo	OFcsc	OFpd
BET surface area (m^2^/g)	17.22 ± 1.23b	17.45 ± 2.40b	21.88 ± 1.08a	24.28 ± 0.20a	22.75 ± 2.86a	15.14 ± 0.95b
Total pore volume (cm^3^/g)	0.07 ± 0.001c	0.06 ± 0.003d	0.07 ± 0.002b	0.08 ± 0.002a	0.07 ± 0.001b	0.05 ± 0.001e
Average pore diameter (μm)	14.02 ± 0.60a	13.02 ± 0.73a	13.83 ± 0.50a	14.30 ± 0.61a	13.84 ± 0.21a	13.21 ± 0.49a

Different lowercase letters accompanying the values indicate statistically significant variations (*p* < 0.05) in physicochemical properties of rhizosphere soils among different TOCS-based organic fertilizers.

**FIGURE 2:**
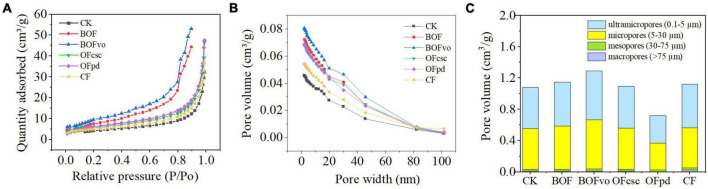
**(A)** Isothermal adsorption curve, **(B)** pore size distribution, and **(C)** pore volume distribution.

Figure 2A demonstrates that, compared to the CK and CF groups, the addition of TOCS-based organic fertilizers enhances the adsorption capacity of rhizosphere soils under identical relative pressure, with BOFvo exhibiting the highest increase followed by BOF, OFpd, and OFcsc. Meanwhile, Figure 2B reveals that, compared to the CK and CF groups, BOFvo significantly improves pore size distribution in the rhizosphere soils. Additionally, Figure 2C illustrates that the soil pore size distribution was primarily within the range of 0.1–30 μm, with ultramicropores, micropores, mesopores, and macropores accounting for average pore volume proportions of 48.66, 48.24, 2.29, and 0.63%, respectively. Compared to the CK and CF groups, the addition of TOCS-based organic fertilizers, particularly with BOFvo treatment, BOFvo significantly increased the average pore volume proportions of ultramicropores, microporosity, and mesoporosity in the rhizosphere soils. These findings indicate that the application of BOFvo has a more significant impact on the pore characteristics of the rhizosphere soils.

As presented in Table 4, the application of TOCS-based organic fertilizers did not result in a significant alteration of soil pH values. However, compared to the CK group, the application of BOFvo led to a substantial increase in TOM, TN, AN, TP, AP, TK, and AK contents of the rhizosphere soils by 20.7, 187.6, 150.1, 264.7, 67.6, 38.8, and 201.5%, respectively. Notably, compared to CF, there was a remarkable enhancement in TOM, TN, AN, TP, TK, and AK contents of the rhizosphere soils by 35.5, 54.2, 22.1, 158.3, 39.6, and 53.8%, respectively. The results suggest that the utilization of BOFvo has a more pronounced influence on the physicochemical characteristics of the rhizosphere soils.

**TABLE 4:** Physicochemical properties of the rhizosphere soils application of different TOCS-based organic fertilizers.

Parameters	CK	CF	BOF	BOFvo	OFcsc	OFpd
pH	4.63 ± 0.16a	4.57 ± 0.05a	4.29 ± 0.11ab	4.82 ± 0.54a	4.36 ± 0.17a	4.29 ± 0.11ab
TOM (%)	35.67 ± 3.47b	31.78 ± 3.68b	41.97 ± 3.14a	43.06 ± 2.88a	35.31 ± 4.43b	33.67 ± 1.44b
TN (g/kg)	0.89 ± 0.18d	1.66 ± 0.08c	2.04 ± 0.03b	2.56 ± 0.11a	2.11 ± 0.11b	2.04 ± 0.03b
AN (mg/kg)	90.39 ± 13.68	185.20 ± 5.01ab	177.77 ± 7.71b	226.05 ± 47.62a	182.34 ± 22.81ab	151.10 ± 18.73b
TP (g/kg)	0.34 ± 0.13c	0.48 ± 0.04c	0.87 ± 0.08b	1.24 ± 0.17a	0.87 ± 0.11b	0.83 ± 0.11b
AP (mg/kg)	2.78 ± 0.47c	7.83 ± 0.92a	4.82 ± 1.01b	4.66 ± 0.71b	3.64 ± 0.32bc	2.15 ± 0.61d
TK (g/kg)	20.19 ± 1.27b	20.07 ± 1.23b	28.04 ± 0.71a	28.02 ± 1.15a	28.08 ± 2.19a	28.04 ± 0.71a
AK (mg/kg)	83.27 ± 24.25c	163.20 ± 32.31b	284.96 ± 11.23a	251.06 ± 36.09a	204.80 ± 15.55b	84.96 ± 7.23c

Different lowercase letters accompanying the values indicate statistically significant variations (*p* ± 0.05) in physicochemical properties of the rhizosphere soils among different TOCS-based organic fertilizers.

### 3.3. Nutrient contents analysis of tea oil camellia leaves

As depicted in Table 5, the application of TOCS-based organic fertilizers had a significant impact on the TN, TP, and TK contents of tea oil camellia leaves compared to CK and CF groups. Significantly, compared to CF, the use of BOFvo resulted in a 40.8 and 186.9% increase in TN and TP contents, respectively, indicating its potential for promoting tea oil camellia growth.

**TABLE 5:** Nutrient contents analysis of tea oil camellia leaves pre- and post-fertilization under different TOCS-based organic fertilizers treatment.

Parameters	CK	CF	BOF	BOFvo	OFcsc	OFpd
TN (g/kg)	6.80 ± 1.65b	7.52 ± 0.46b	11.32 ± 1.85a	10.59 ± 1.15a	9.14 ± 1.40a	11.29 ± 0.29a
TP (g/kg)	0.70 ± 0.57d	0.99 ± 0.12d	2.15 ± 0.08b	2.84 ± 0.16a	1.51 ± 0.03c	1.11 ± 0.07d
TK (g/kg)	5.82 ± 0.20c	7.54 ± 2.24b	9.06 ± 0.51a	7.49 ± 0.27b	9.54 ± 0.83a	6.14 ± 0.24c

Different lowercase letters indicate significant differences (*p* < 0.05) in nutrient contents of the tea oil camellia leaves among different TOCS-based organic fertilizer treatments.

### 3.4. Effect on fruit yields of tea oil camellia

As shown in Table 6, compared to CK and CF groups, the application of TOCS-based organic fertilizers had a significant impact on the average yield and fruiting rate of individual plant at the mature stage. Significantly, in comparison to CK and CF group, BOFvo treatment resulted in a significant increase of 115.0, 35.4 and 252.8, 128.7% in average yield and fruiting rate, respectively, indicating its potential for enhancing the fruit yields of tea oil camellia.

**TABLE 6:** The average yield and fruiting rate of individual plant at the mature stage.

Treatments	Average yield (kg)	Fruiting rate (%)
CK	1.42 ± 1.08c	16.04 ± 3.78c
CF	2.33 ± 0.35b	27.09 ± 5.26b
BOF	3.59 ± 1.14ab	32.36 ± 0.64ab
BOFvo	5.01 ± 1.41a	36.69 ± 2.32a
OFcsc	2.73 ± 0.37b	25.33 ± 5.23b
OFpd	2.37 ± 0.66b	19.02 ± 9.10c

Different lowercase letters accompanying the values indicate statistically significant variations (*p* < 0.05) in average yield and fruiting rate of individual plant at the mature stage among different TOCS-based organic fertilizers treatment.

### 3.5. Sequences data

As shown in Table 7, with respect to the sequencing data of bacteria in the rhizosphere soils, after cleaning and quality checking, 79,788, 79,732, 79,991, 79,772, and 79,794 clean reads were generated from CK, BOF, BOFvo, OFcsc, and OFpd treatment, respectively. Analyses of the OTUs revealed that 64,418, 58,466, 58,661, 59,311, and 63,486 OTUs were shared by CK, BOF, BOFvo, OFcsc, and OFpd treatment, respectively. In the meantime, CK, BOF, BOFvo, OFcsc, and OFpd treatments yielded 79,625, 79,828, 74,395, 60,150, and 79,576 clean reads as well as 75,665, 74,843, 64,802, 48,146, and 73,073 OTUs. It is noteworthy that the coverage exceeded over than 99.0%, indicating satisfactory numbers of analyzed OTUs with most bacteria and fungi present in soil samples being detected.

**TABLE 7:** Overview of sequencing data of bacteria and fungi in the rhizosphere soils under different TOCS-based organic fertilizer treatment.

Treatment	Bacteria				Fungi			
	Raw reads	Clean reads	OTUs	Coverage (%)	Raw reads	Clean reads	OTUs	Coverage (%)
CK	80,016	79,788	64,418	99.98	79,900	79,625	75,665	99.98
BOF	79,908	79,732	58,466	99.99	80,179	79,828	74,843	99.99
BOFvo	80,168	79,991	58,661	99.98	74,690	74,395	64,802	99.98
OFcsc	79,974	79,772	59,311	99.99	60,229	60,150	48,146	99.99
OFpd	79,956	79,794	63,486	99.98	79,888	79,576	73,073	99.98

### 3.6. Effects on microbial diversity in the rhizosphere soils

The alpha-diversity of bacterial and fungal communities in the rhizosphere soils was assessed by computing the ACE, Chao1, and Shannon indices. Table 8 reveals that for bacteria, the Chao1, ACE, and Shannon indices were significantly higher in BOFvo treated soils (exhibiting an increase of 85.1, 78.0, and 12.0%) compared to CK group. For fungi, Table 8 also shows that, compared with CK group, the Chao1 and ACE indices in BOFvo treatment (increased by 13.1, and 11.1%, respectively) were significantly increased. The results indicate that the incorporation of TOCS-based organic fertilizers, particularly BOFvo, significantly enhances the microbial diversity in the rhizosphere soils.

**TABLE 8:** The alpha-diversity indices of bacteria and fungi in the rhizosphere soils under different TOCS-based organic fertilizer treatment.

Treatment	Bacteria			Fungi		
	Chao1	ACE	Shannon	Chao1	ACE	Shannon
CK	998.30 ± 0.07d	995.90 ± 7.49d	8.3 ± 0.15b	557.8 ± 8.64c	568.5 ± 5.48b	6.3 ± 0.99b
BOF	1,632.80 ± 10.77b	1,456.20 ± 3.58c	9.4 ± 0.25a	627.4 ± 6.84b	628.0 ± 6.65a	7.4 ± 0.65a
BOFvo	1,847.70 ± 9.64a	1,773.10 ± 10.95a	9.3 ± 0.90a	631.0 ± 3.36b	631.6 ± 3.14a	6.1 ± 0.58b
OFcsc	1,673.10 ± 8.19b	1,677.30 ± 9.12b	9.3 ± 0.34a	558.9 ± 8.80c	630.9 ± 9.84a	6.2 ± 0.75b
OFpd	1,303.00 ± 4.23c	1,009.50 ± 2.77d	7.6 ± 1.83b	698.5 ± 6.16a	566.1 ± 6.87b	6.4 ± 0.52b

Different lowercase letters accompanying the values indicate that diversity and richness indices of bacteria and fungi with significant difference at *p* < 0.05 among different TOCS-based organic fertilizers.

### 3.7. PCA of microbial communities in the rhizosphere soils

**FIGURE 3:**
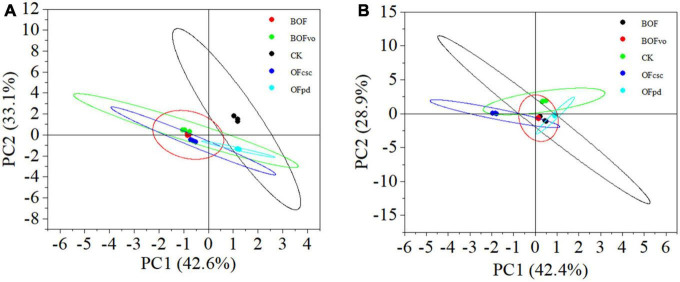
Principal component analysis of microbial communities at genus level in the rhizosphere soil. **(A)** PCA of bacterial community at genus level. **(B)** PCA of fungal community at genus level.

To explore the diversity of microbial communities in the rhizosphere soils treated with different TOCS-based organic fertilizers, we conducted PCA at the genus level to evaluate bacterial and fungal diversity. The PCA results for bacterial diversity (Figure 3A) shows that the five rhizosphere soil samples were distributed across different quadrants based on PC1 accounting for 42.6% and PC2 accounting for 33.1%, indicating large differences in the soil bacterial communities with different TOCS-based organic fertilizers treatments. The bacterial community in the rhizosphere soil treated with BOFvo differed significantly from those treated with CK, BOF, OFpd, and OFcsc. Meanwhile, the results of PCA analysis on fungal diversity (Figure 3B) reveals that the five rhizosphere soil samples were distributed in different quadrants based on PC1 accounting for 42.4% and PC2 accounting for 28.9%, indicating significant differences in the soil fungal community among various TOCS-based organic fertilizer treatments. Notably, compared to CK, BOF, BOFvo, and OFpd treatments, OFcsc treatment significant differences were observed at the genus level of fungal community in rhizosphere soil. In summary, the application of TOCS-based organic fertilizers exerted a significant influence on the microbial communities in the rhizosphere soil.

### 3.8. Effects on the microbial relative abundances and community structure in the rhizosphere soils

**FIGURE 4:**
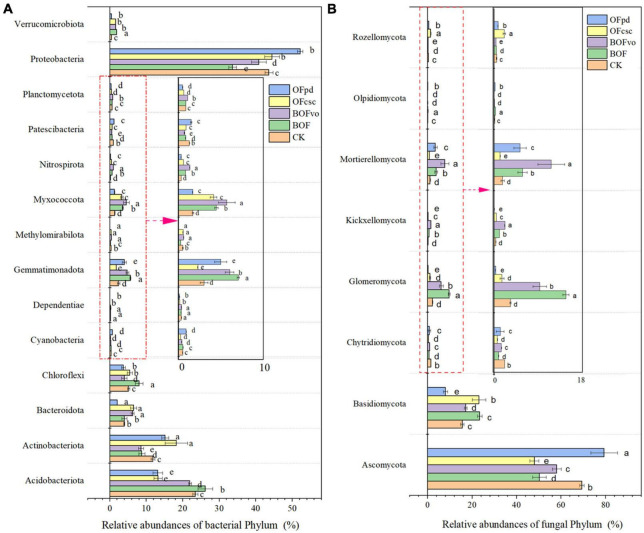
Microbial relative abundances at the phylum level in the rhizosphere soils treated by different TOCS-based organic fertilizers. **(A)** Relative abundances of the bacterial at the phylum level. **(B)** Relative abundances of the fungal at the phylum levels. Different lowercase letters indicate that the relative abundances of fungal and bacteria phylum in the rhizosphere soils with significant difference at *p* < 0.05 among different TOCS-based organic fertilizers treatment.

The impact of TOCS-based organic fertilizers on the relative abundance of bacterial and fungal phyla in the rhizosphere soils was assessed and the results were depicted in Supplementary Tables 1, 2 and Figure 4. According to the sequencing detected, a total of 30 bacteria (Supplementary Table 1) and 15 fungi phyla (Supplementary Table 2) were detected and classification in the all rhizosphere soils samples. As shown in Supplementary Table 1 and Figure 4A, Proteobacteria was identified as the dominant bacterial phyla present in the rhizosphere soils, followed by Acidobacteriota and Actinobacteriota. According to the taxonomic analysis, after BOF treatment, the relative abundances of Verrucomicrobiota, Nitrospirota, Myxococcota, Gemmatimonadota, Cyanobacteria, Chloroflexi, Bacteroidota, and Acidobacteriota in the rhizosphere soils significantly increased. After BOFvo treatment, the relative abundances of Verrucomicrobiota, Planctomycetota, Nitrospirota, Myxococcota, Methylomirabilota, Gemmatimonadota, and Bacteroidota in the rhizosphere soils significantly increased. Meanwhile, OFcsc treatment could significantly increase the relative abundances of Verrucomicrobiota, Proteobacteria, Nitrospirota, Myxococcota, Methylomirabilota, Chloroflexi, Bacteroidota, and Acidobacteriota in the rhizosphere soils. In addition, OFpd treatment significantly increased the relative abundances of Proteobacteria, Patescibacteria, Methylomirabilota, Gemmatimonadota, Cyanobacteria, and Actinobacteriota in the rhizosphere soils. Supplementary Table 2 and Figure 4B illustrates that the dominant fungal phylum was Ascomycota in the rhizosphere soils, followed by Basidiomycota, its relative abundance decreased after the addition of BOF, BOFvo, and OFcsc, while increased after the addition of OFpd. The relative abundances of Basidiomycota, Glomeromycota, and Mortierellomycota significantly increased with the BOF and BOFvo treatments. The OFcsc treatment significantly increased the abundances of Basidiomycota and Zoopagomycota, but decreased the abundances of Basidiomycota, Glomeromycota, and Kickxellomycota in the OFpd treatment.

**FIGURE 5:**
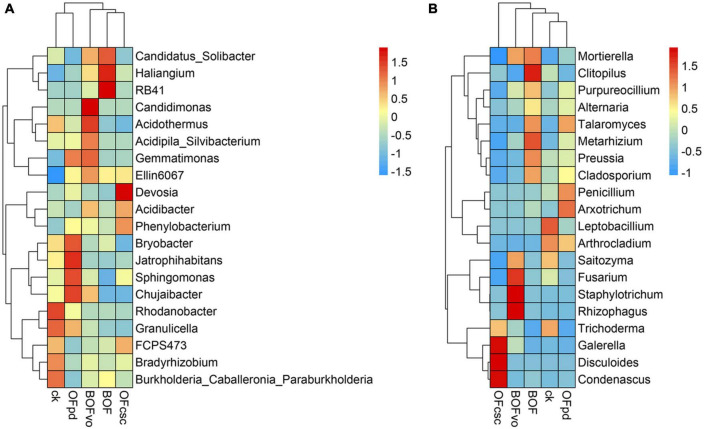
The heatmap of bacterial and fungal genus abundances in the rhizosphere soils treated by different TOCS-based organic fertilizers. **(A)** Spearman’s correlations between the bacterial genera abundances in the rhizosphere soils and different TOCS-based organic fertilizers. **(B)** Spearman’s correlations between the fungal genera abundances in the rhizosphere soils and different TOCS-based organic fertilizers.

The correlation between TOCS-based organic fertilizers and the structure of microbial communities was analyzed, with the findings presented in Supplementary Tables 3, 4 and Figure 5. According to the sequencing detected, a total of 466 bacteria (Supplementary Table 3) and 532 fungi genera (Supplementary Table 4) were detected and classification in all rhizosphere soils samples. Supplementary Table 3 also illustrates that the most dominant bacterial genus was *Rhodanobacter*, followed by *Acidothermus*, *Sphingomona*s, *Acidibacter*, *Bryobacter*, *Burkholderia_Caballeronia_Paraburkholderia*, and *Candidatus Solibacter*. Meanwhile, Supplementary Table 4 illustrates that the dominant fungal genus was *Fusarium*, followed by *Leptobacillium*, *Trichoderma*, *Penicillium*, *Saitozyma*, and *Arthrocladium*. As shown in Supplementary Table 3 and Figure 5A, compared to CK group, the abundance of bacterial genera significantly increased the abundance of *Candidatus Solibacter*, *Candidimonas*, *Acidothermus*, *Acidipila Silvibacterium*, *Gemmatimonas*, *Ellin 6067*, *Acidibacter*, and *Chujaibacter* with the BOFvo treatment; *Gemmatimonas*, *Bryobacter*, *Jatrophihabitans*, *Sphingomonas*, *Chujaibacter*, and *Granulicella* with the OFpd treatment; *Candidatus Solibacter*, *Haliangium*, *RB41*, *Ellin 6067*, *Bryobacter*, and *Burkholderia Caballeronia Paraburkholderia* with the BOF treatment; and *Devosia*, *Acidibacter*, *Phenylobacterium*, and *FCPS473* with the OFcsc treatment. Supplementary Table 4 and Figure 5B shows that the abundance of fungal genera significantly increased the abundance of *Fusarium*, *Mortierella*, *Saitozyma*, *Staphylotrichum*, and *Rhizophagus* with the BOFvo treatment; *Clitopilus*, *Metarhizium*, *Mortierella*, *Preussia*, *Purpureocillium*, *Cladosporium*, and *Talaromyces* with the BOF treatment; *Condenascus*, *Disculoides, Galerella*, and *Trichoderma* with the OFcsc treatment; and *Arthrocladium*, *Arxotrichum, Penicillium*, and *Talaromyces* in OFpd treatments.

### 3.9. Relationship between the physicochemical properties of TOCS-based organic fertilizers and microbial communities of the rhizosphere soils

Redundancy analysis was employed to evaluate the associations between the physicochemical properties of different TOCS-based organic fertilizers and microbial communities (at a phylum level) of the rhizosphere soils. Table 9 shows that the pore volume (contribution rate 36.6%) and TP (contribution rate 36.4%) had the most significant effect on the bacterial community structure, followed by AP, pH, ultramicropore, surface, and TN. Among of them, pore volume, TP, AP, pH, ultramicropore, and surface significantly affected the bacterial community of the rhizosphere soils. Meanwhile, pore volume, TP, AP, pH, and surface significantly affected the fungal community, with the greatest effect by the surface (contribution rate 72.2%), followed by TK, ultramicropore, AP, TP, and pH, which shows that soil pore structure was the main factor affecting the fungal community of the rhizosphere soils.

**TABLE 9:** Redundancy analysis results showing the contribution and statistical significance.

Parameters	Bacterial			Fungal		
	Contribution rate (%)	Pseudo-F	*p*-Value	Contribution rate (%)	Pseudo-F	*p*-Value
Pore volume	36.6	7.5	0.014	0.5	6.4	0.028
TP	36.4	16.1	0.002	1.7	3.6	0.004
AP	10.0	6.5	0.010	3.6	4.2	0.024
pH	7.3	7.5	0.006	3	4.7	0.008
Ultramicropore	2.8	3.6	0.022	4.7	4.2	0.060
Surface	2.2	3.8	0.046	72.2	33.7	0.002
TN	1.3	2.6	0.110	<0.1	2.1	0.320
AK	0.9	2.2	0.146	0.7	3.7	0.064
AN	0.4	0.9	0.434	1	3.4	0.064
Macropore	0.3	0.6	0.516	<0.1	1.3	0.358
Micropore	0.1	0.3	0.664	1	2.4	0.144
TOM	0.2	0.2	0.702	<0.1	<0.1	1.00
Diameter	0.5	0.5	0.624	0.6	1.5	0.266
Mesopore	1.0	<0.1	1.00	<0.1	<0.1	1.00
TK	<0.1	<0.1	1.00	11.0	7.8	0.002

**FIGURE 6:**
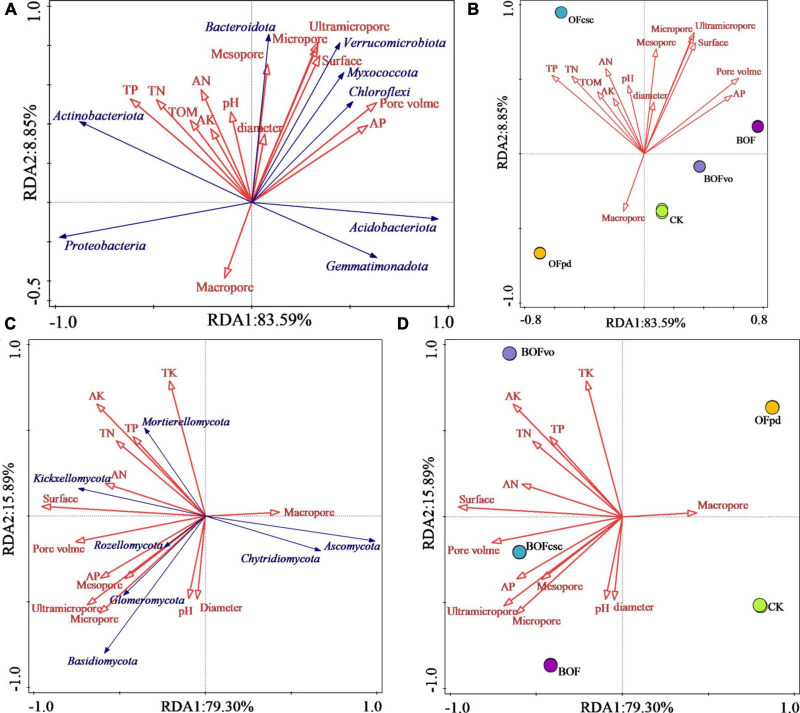
**(A,B)** Redundancy analysis between the physicochemical properties of TOCS-based organic fertilizers and bacterial community of the rhizosphere soils. **(C,D)** RDA between the physicochemical properties of TOCS-based organic fertilizers and fungal community of the rhizosphere soils.

As shown in Figure 6, the RDA PC1 and PC2 accounted for 92.44% (PC1 = 83.59% and PC2 = 8.85%) and 95.19% (PC1 = 79.30%, PC2 = 15.89%) of the bacteria and fungi total variation, respectively. Figures 6A, B demonstrate that the physicochemical properties of the rhizosphere soil, including TP, TN, TA, AN, TOM, AK, pore volume, ultramicropore, micropore diameter, and mesopore, have a positive impact on the abundance of bacterial community, such as Actinobacteriota, Bacteroidota, Verrucomicrobiota, Myxococcota, and Chloroflexi, conversely, macropore had a positive impact on the abundance of Proteobacteria, Acidobacteriota, and Gemmatimonadota. Meanwhile, Figures 6C, D show that Basidiomycota, Rozellomycota, Glomeromycota, Kickxellomycota, and Mortierellomycota were positively influenced by TK, TP, TN, AK, AN, AP, pH, TOM, ultramicropore, micropore, pore volume, and surface of the rhizosphere soil, conversely, Chytridiomycota and Ascomycota were primarily affected by the macropore of the rhizosphere soil. Therefore, the RDA results reveal that the physicochemical characteristics of TOCS-based organic fertilizers had a significant impact on the composition and distribution of microbial communities in the rhizosphere soils.

## 4. Discussion

Soil microorganisms play a crucial role in promoting the restoration of soil ecosystems. However, current research has mainly focused on changes in microbial community structure, with little attention given to soil physicochemical properties and their relationship with microbial communities. In this study, we prepared four types of TOCS-based organic fertilizers and evaluated their effects on the physicochemical property and microbial community structure of the rhizosphere soils. The by-product of tea oil camellia processing, known as TOCS, is typically discarded or incinerated due to the lack of feasible reuse techniques. As a result, TOCS is primarily disposed of through incineration or landfill methods, leading to various forms of pollution such as groundwater and soil contamination, as well as greenhouse gas emissions (Su et al., 2014; Qin et al., 2018). The utilization of TOCS not only enhances its own value but also addresses the issue of environmental pollution caused by it. As a valuable biomass resource, TOCS contains tea saponin, polysaccharides, tannins, as well as protein, nitrogen, phosphorus, and potassium. These components can be composted into an effective organic fertilizer to enhance crop yield and quality (Yang et al., 2015). As a rich waste biomass resource, TOCS contains not only tea saponin, polysaccharide, tannin, but also protein, nitrogen, phosphorus, potassium and so on, which can be composted into an efficient organic fertilizer to improve crop yield and quality (Samuel et al., 2018; Sheoran et al., 2019; Anyega et al., 2021; Liu et al., 2021). Application of bio-organic fertilizers in farmland can enhance the physical and chemical properties, regulate the composition of soil microbial community, and promote healthy and vigorous crop growth by increasing the richness and uniformity of beneficial microbial communities (Syed et al., 2021). In order to mitigate pollution, conserve non-renewable energy resources, and foster ecological civilization, it is imperative to fully leverage TOCS and generate greater value.

Characterizing and quantifying changes in soil pore structure, a critical indicator for understanding the impact of fertilization on soil quality, is essential to determining the effects of organic fertilizers on soil structure and hydraulic properties (Lu et al., 2019). Soil porosity facilitates a range of functions and processes, including nutrient transportation, water and gas storage, as well as microbial activity (Kong et al., 2016). Fan et al. (2020) discovered that the application of biochar in a wheat-rice rotation soil resulted in decreased porosity, but improved macropores and pore connectivity. Additionally, the geometry of soil pores has been demonstrated to be linked to the diversity and activity of microbial communities (Bhattacharyya et al., 2021; Yang et al., 2022). Our findings suggest that the utilization of TOCS-based organic fertilizers can enhance the BET surface area and total pore volume of the rhizosphere soils, which is in line with the outcomes reported by Singh et al.’s (2021) investigation, indicating that soil porosity was significantly improved by applying cattle manure. The presence of fine soil particles that fill the larger pores indicates that the interaction between soil particles and macropores in fertilizers plays a crucial role in modifying pore structure (Wang et al., 2019; Yang et al., 2021).

The growth and reproduction of soil microorganisms are significantly influenced by various environmental factors, including but not limited to soil depth, moisture content, organic matter concentration, porosity, oxygen and carbon dioxide levels, as well as soil pH (Cao et al., 2016). Fertilization has the potential to enhance soil physical and chemical properties, which in turn can influence microbial community composition and activity, ultimately improving microorganism habitat quality (Liu et al., 2010; Zhang et al., 2022). Our findings demonstrate that the application of TOCS-based organic fertilizers did not lead to a significant alteration in soil pH values, however, it exerted a more pronounced influence on the levels of TOM, TN, AN, TP, AP, TK, and AK in the rhizosphere soils, which is in line with the reported by Wu et al. (2021). Meanwhile, the results of our study also demonstrate that the application of TOCS-based organic fertilizers significantly influenced the nutrient (TN, TP, and TK) levels in tea oil camellia leaves, thereby indicating its potential for promoting tea oil camellia growth to enhance the fruit yields of tea oil camellia. Some previous studies had demonstrated that application of organic fertilizers could improve the fruit quality, nutritional value and yields (Marzouk and Kassem, 2011; Khaitov et al., 2019; Negi et al., 2021).

Organic manures are recognized for their ability to enhance the stability of rhizosphere microbial communities and associated functions (Syed et al., 2021). Previous researches have demonstrated that organic fertilizers have a positive impact on soil microbial alpha-diversity indices (Shannon, Chao, and ACE), promoting the reproduction and growth of microbial populations while increasing overall diversity (Ding et al., 2018; Ji et al., 2018; Wu et al., 2021). The findings of our study suggest that the application of TOCS-based organic fertilizers significantly enhances the ACE, Chao1, and Shannon indices, thereby promoting microbial diversity in the rhizosphere soils. Meanwhile, PCA at the genus level also suggests significant impact of application of different TOCS-based organic fertilizers on the soil microbial diversity. This is primarily due to differences substrate utilization in different TOCS-based organic fertilizers. The findings of Bucher and Lanyon (2005) demonstrated that the application of dairy manure annually or biennially resulted in distinct soil microbial communities compared to soils without manure or with occasional applications.

Our findings suggest that the application of TOCS-based organic fertilizers significantly enhances the microbial diversity in rhizosphere soils with Proteobacteria and Ascomycota being the dominant bacterial and fungal phyla, respectively, and *Rhodanobacter* and *Fusarium* being the dominant bacterial and fungal genus, respectively. *Proteobacteria* is an evolutionarily, geologically, and environmentally important group of microorganisms. Our findings indicate that *Proteobacteria* is the predominant bacterial phylum in rhizosphere soils treated with TOCS-based organic fertilizers. This aligns with previous studies which have also identified Proteobacteria as the most abundant genus in soil libraries due to its vast morphological, physiological, and metabolic diversity, and crucial role in global nitrogen, sulfur, and carbon cycling (Janssen, 2006; Kersters et al., 2006). Likewise, despite the fact that Proteobacteria is the most abundant genus in soil and has more validly described species than any other phyla, the functions of most Proteobacteria in soil have yet to be demonstrated. Meanwhile, our findings have also indicated that Ascomycota dominates the rhizosphere soils treated with TOCS-based organic fertilizers. Similar results from previous studies have identified Ascomycota as the predominant fungal phylum in soil systems worldwide (Maestre et al., 2015; Egidi et al., 2019). *Rhodanobacter* participates in the soil nitrogen cycle by means of denitrification (Green et al., 2012; Kostka et al., 2012). In this study, *Rhodanobacter* was identified as the most dominant bacterial genus, which is consistent with Wang et al.’s (2017) report that *Rhodanobacter* dominated the bacterial distribution at the genus level in acclimated sludge. However, our findings indicate that the application of TOCS-based organic fertilizers may increase the risk of wilt, blight, rot and canker in plants due to the dominance of *Fusarium*, a habitat for all organisms involved in soil-borne diseases (Orr and Nelson, 2018), as the bacterial genus present in the rhizosphere soils treated with such TOCS-based organic fertilizers.

## 5. Conclusion

The current study involved the preparation of four organic fertilizers based on TOCS. Our findings provide evidence that the application of TOCS-based organic fertilizers significantly improves soil physicochemical properties and microbial community structure in the rhizosphere soil, as well as nutrient contents in tea oil camellia leaves and average yield and fruiting rate per plant at maturity. In addition, our forthcoming research will concentrate on the long-term sustainability and impact of TOCS-based organic fertilizers on soil fertility, plant health, and ecosystem dynamics. This study will facilitate the promotion and application of TOCS-based organic fertilizers, thereby establishing a foundation for the reuse of tea oil camellia waste resources.

## Data availability statement

The data presented in the study are deposited in the National Center for Biotechnology Information (NCBI) repository, accession numbers PRJNA999865 and PRJNA999913.

## Author contributions

AH: methodology and writing—original draft preparation. ZW: software. DY and WB: validation. NW and XL: investigation. ZL: data curation. AH and SY: writing—review and editing and project administration. SY: funding acquisition. All authors had read and agreed to the published version of the manuscript.

## Footnotes

1
http://www.metaboanalyst.ca

2
http://www.canoco5.com/

## Supplementary material

Supplementary Table 1 The relative abundance of bacterial phyla in the rhizosphere soils.

Supplementary Table 2 The relative abundance of fungal phyla in the rhizosphere soils.

Supplementary Table 3 The relative abundance of bacterial genus in the rhizosphere soils.

Supplementary Table 4 The relative abundance of fungal genus in the rhizosphere soils.
